# The effect of carnosine or β-alanine supplementation on markers of glycaemic control and insulin resistance in human and animal studies: a protocol for a systematic review and meta-analysis

**DOI:** 10.1186/s13643-020-01539-8

**Published:** 2020-12-05

**Authors:** Joseph J. Matthews, Eimear Dolan, Paul A. Swinton, Livia Santos, Guilherme G. Artioli, Mark D. Turner, Kirsty J. Elliott-Sale, Craig Sale

**Affiliations:** 1 Sport, Health and Performance Enhancement (SHAPE) Research Centre, Musculoskeletal Physiology Research Group, School of Science and Technology, Nottingham Trent University, Nottingham, UK; 2 Research Centre for Life and Sport Sciences (CLaSS), School of Health and Life Sciences, Department of Sport and Exercise, Birmingham City University, Birmingham, UK; 3 Applied Physiology and Nutrition Research Group, Rheumatology Division, Faculdade de Medicina FMUSP, Universidade de São Paulo, São Paulo, Brazil; 4 School of Health Sciences, Robert Gordon University, Aberdeen, UK; 5 Centre for Diabetes, Chronic Diseases, and Ageing, School of Science and Technology, Nottingham Trent University, Nottingham, UK

**Keywords:** Diabetes, Prediabetes, Metabolic health, Glucose, Therapeutics, Nutrition

## Abstract

**Background:**

Diabetes is a major public health issue and there is a need to develop low-cost, novel interventions to prevent or reduce disease progression. Growing evidence shows that supplementation with carnosine, or its rate-limiting precursor β-alanine, can ameliorate aspects of the metabolic dysregulation that occurs in diabetes. There is, however, a need to develop a better understanding of the magnitude of effect and the factors associated with positive outcomes. The purpose of this systematic review and meta-analysis is to evaluate the effect of carnosine or β-alanine supplementation on markers of glycaemic control and insulin resistance in humans and animals.

**Methods:**

We will perform a systematic search for randomised and non-randomised controlled trials. Studies will be retrieved by searching electronic databases, clinical trial registers, author review, and cross-referencing. Primary outcomes include changes in (i) fasting glucose, (ii) glycated haemoglobin, and (iii) 2-h glucose following a glucose tolerance test. A set of additional outcomes includes other markers of glycaemic control and insulin resistance. Risk of bias (RoB) will be assessed using the Cochrane RoB 2.0 tool (human studies) and the Systematic Review Centre for Laboratory Animal Experimentation (SYRCLE) RoB tool (animal studies). Confidence in the cumulative evidence will be assessed using the Grading of Recommendations Assessment, Development and Evaluation (GRADE) approach. All meta-analyses will be conducted within a Bayesian framework, providing a flexible modelling approach to account for uncertainty in model parameters and underlying structures within the data.

**Discussion:**

By including all available human and animal data, we will provide the most comprehensive overview on the topic to date. The results will have implications for those working in prediabetes, diabetes, and metabolic health in general and may lead to the development of new treatment approaches.

**Dissemination:**

Study results will be presented at a professional conference and published in a peer-reviewed journal.

**Systematic review registration:**

CRD42020191588

**Supplementary Information:**

The online version contains supplementary material available at 10.1186/s13643-020-01539-8.

## Background

Diabetes is a major public health problem. Recent estimates show that 4.8 million people in the UK are living with diabetes, which is expected to rise to 5.3 million by 2025 [1]. Type 2 diabetes accounts for over 90% of these cases, with the remaining made up of type 1 diabetes, gestational diabetes, and rarer types of diabetes (e.g. maturity onset diabetes of the young). A hallmark of type 2 diabetes is poor glycaemic control and insulin resistance [2], which can present earlier in life as impaired fasting glucose or impaired glucose tolerance (also known as prediabetes). For individuals aged 45 years, there is a 74% lifetime risk of progression from prediabetes to type 2 diabetes [3]. While lifestyle modifications are central to risk reduction, they can be challenging to implement, and long-term adherence limits their effectiveness. It is therefore important to develop low-cost, novel interventions to improve glycaemic control and help prevent or delay disease progression.

The multifunctional compound carnosine has emerged as a candidate for clinical use. Carnosine, a member of the histidine-containing dipeptide (HCD) family, exists naturally in high concentrations in mammalian skeletal muscle and in smaller amounts in other excitable tissues [4–7]. Contents in skeletal muscle can be increased by supplementing with carnosine or its rate-limiting precursor β-alanine [8]. Work from our Research Group shows that treatment with carnosine decreases highly toxic lipid peroxidation products in skeletal muscle cells (unpublished data), leading to an increase in insulin-stimulated glucose uptake under glucolipotoxic conditions [9]. Further evidence supports the role of carnosine in non-enzymatic detoxification of reactive aldehydes [10, 11], an effect that β-alanine supplementation potentiates in vivo [12, 13]. Through these actions, carnosine may be able to ameliorate aspects of the metabolic dysregulation that occurs in diabetes and its related conditions.

There is growing evidence from rodent studies that carnosine supplementation can prevent or delay the development of type 2 diabetes [14, 15]. Initial human trials also show promise [16, 17], but the factors associated with positive outcomes are unclear. A recent meta-analysis of human studies sought to address this knowledge gap, concluding that supplementation with histidine-containing dipeptides improves waist circumference, fasting glucose, and glycated haemoglobin (HbA_1c_) [18]. The review, however, had several methodological shortcomings (for details, see [19]), which included combining effects from studies using multi-ingredient supplements with those supplementing carnosine or β-alanine in isolation. This approach cannot determine whether the beneficial effects are due to carnosine or β-alanine alone. It is also important to consider relevant outcomes from animal models that can provide mechanistic insight and help inform future human research studies. There is a clear need to develop a better understanding of the magnitude of effect, as well as the factors associated with supplementation for improving outcomes. Therefore, the purpose of this systematic review and meta-analysis is to evaluate the effect of carnosine or β-alanine supplementation on markers of glycaemic control and insulin resistance in humans and animals.

## Methods

This protocol follows the Preferred Reporting Items for Systematic Review and Meta-Analysis Protocols (PRISMA-P) [20] and is registered on PROSPERO (registration number CRD42020191588).

### Eligibility criteria

Studies will be selected according to the eligibility criteria outlined in Table 1. There will be no restrictions on the timing or duration of supplementation and no restrictions on the type of setting. English and non-English language sources will be included with the latter translated into English using freely available online translators (e.g. Google or Bing). Any studies that cannot be adequately translated will be excluded from the review and a list of the titles provided as an appendix.

**Table 1:** Overview of PICOS eligibility criteria

Participants	Humans with a diagnosis of type 1 diabetes, type 2 diabetes, prediabetes, gestational diabetes, impaired fasting glucose, or impaired glucose tolerance (according to WHO guidelines [21, 22]), or humans with overweight/obesity (BMI ≥ 25 kg^.^m^2^) where the relevant outcomes were collected and reported. Animal studies using a diabetes-related disease model (see human criteria above), or overweight/obese animals where the relevant outcomes were collected and reported. There will be no restriction on age or comorbidities and no restrictions on the methods used to induce disease in animal studies.
Intervention	Supplementation with carnosine or β-alanine. We will exclude studies that use a multi-ingredient supplement intervention. Human studies will include oral administration only, whereas in animal studies we will also consider administration by other means (e.g. intraperitoneal or intravenous injection).
Comparator	Comparisons for human studies will be between placebo and the experimental intervention. Comparisons for animal studies will be between placebo or control (no intervention) and the experimental intervention. Studies without a control or placebo group will be excluded.
Outcomes	Outcomes relating to glycaemic control and insulin resistance. Primary outcomes include changes in (i) fasting glucose, (ii) glycated haemoglobin, and (iii) 2-h glucose following a glucose tolerance test. Additional outcomes include changes in fasting insulin, glucose tolerance test parameters, and homeostatic model assessment parameters (see supporting information for a full list).
Study designs	Studies will be limited to non-randomised and RCTs, including cluster RCTs. We will exclude cohort studies, cross-sectional studies, case series, case reports, commentary, and review articles.

*BMI* body mass index, *RCTs* randomised controlled trials, *WHO* World Health Organization

### Information sources

We will search the following electronic databases for potentially eligible studies: PubMed, Scopus, Web of Science, Cochrane Central Register of Controlled Trials (CENTRAL), Cumulative Index to Nursing and Allied Health Literature (CINAHL), and ProQuest. The electronic database search will be supplemented by searching for trial protocols listed on trial registers: Clinical Trials (www.ClinicalTrials.gov), EU Clinical Trials Register (www.clinicaltrialsregister.eu), International Standard Randomised Controlled Trial Number (ISRCTN) (www.isrctn.com/mrct), and Animal Study Registry (www.animalstudyregistry.org). To ensure full coverage of the literature, we will search the reference lists and perform citation tracking of included studies and relevant reviews identified through the search. Review authors will also search their own personal files to identify any potentially relevant material.

### Search strategy

Search strategies will be developed using key text words and Medical Subject Headings (MeSH) related to the population, intervention, and outcomes (Table 1). A preliminary search strategy for PubMed can be viewed in the supporting information. The search strategy will be peer-reviewed by an academic librarian, not otherwise associated with the project, using the Peer Review of Electronic Search Strategies (PRESS) approach [23]. This search strategy will then be adapted for other databases. Searches will be performed from the earliest record in each database up to the present day and repeated prior to submission of the final review to retrieve any articles published during the interim period.

Initial searches, data extraction, and assessment of risk of bias steps will be completed independently by two reviewers. Full-text screening will be completed independently by three reviewers. Any disagreements will be resolved via consensus-based discussion, and remaining disagreements for searches, data extraction, and risk of bias will be referred to a third reviewer who will provide a recommendation.

### Study records

Titles and abstracts of articles from the initial searches will be imported into a systematic review management platform (Covidence, Veritas Health Innovation Ltd., Melbourne; Australia), duplicates removed, and remaining articles screened for potential eligibility. We will obtain full texts for all articles that appear to meet the inclusion criteria or where there is any uncertainty. Each reviewer will use the reference manager functions to highlight eligibility criteria and add comments on each article, which allows decisions to be cross-referenced in the event of a disagreement. Reviewers will not be blinded to the journal titles or the study authors.

Multiple reports of the same study will be handled by including the published article that provides the most relevant outcome data, assuming similar methods and sample sizes. We will seek additional information from study authors where necessary to resolve issues regarding eligibility (maximum of three e-mail attempts). A PRISMA flow diagram that depicts the search process will be included (see supporting information for a template), as well as supporting information that includes a reference list of all full-text studies excluded, including reasons for exclusion.

Data will be extracted using a standardised spreadsheet based on the Cochrane data collection form for intervention reviews [24]. To ensure consistency between reviewers, we will conduct calibration exercises before starting data extraction.

### Data items

We will extract data for (i) study characteristics (location, setting, study design, size, duration, funding sources, and study aim), (ii) human participant characteristics (age, height, sex, body mass, body mass index, body fat %, type and duration of condition, activity and exercise levels, and dietary information), (iii) animal characteristics (age, body mass, source, species, strain, sex, developmental stage, genetic modification status, genotype, type and duration of condition, method used to induce disease, and housing conditions), (iv) intervention characteristics (name, type of control used, dosage, frequency, duration, route of administration), and (v) outcome characteristics for glycaemic control and insulin resistance (type of measure, sample sizes, baseline, interim, and post-intervention measures of central tendency and dispersion, adherence to the intervention, dropouts, number and nature of side effects, and assessment of blinding to the intervention). We will also extract information relevant to measures of risk of bias and quality assessment. Where necessary, measures of central tendency and dispersion will be extracted from figures in the articles using WebPlotDigitizer version 3.10 (https://apps.automeris.io/wpd/). If parameters cannot be adequately calculated, we will contact study authors for additional data (maximum of three e-mail attempts).

### Outcomes and prioritisation

The three primary outcomes will be changes in fasting glucose (FG) (includes plasma, serum, and blood glucose values), glycated haemoglobin (HbA_1c_), and 2-h glucose following a glucose tolerance test (GTT). These outcomes represent the three clinical markers used in the diagnosis of type 1 diabetes, type 2 diabetes, prediabetes, and gestational diabetes [21, 22]. Additional outcomes include changes in other markers of glycaemic control and insulin resistance (for a full list see supporting information).

### Risk of bias in individual studies

Risk of bias in individual human studies will be assessed using the Cochrane risk of bias 2.0 tool (RoB 2.0) for assessing risk of bias in randomised trials [25] and in individual animal studies using the Systematic Review Centre for Laboratory Animal Experimentation (SYRCLE) tool [26]. Reviewers will assess each study item as either “high risk”, “low risk”, “some concerns” (RoB 2.0), or “unclear risk” (SYRCLE) of bias. All disagreements and referrals will be recorded.

### Data synthesis

Data will be presented in summary tables to describe the study population and intervention. We will conduct meta-analyses using appropriate models to account and explore for variation within and between studies. All meta-analyses will be conducted within a Bayesian framework, providing a flexible modelling approach to account for uncertainty in model parameters and underlying structures within the data. Additionally, Bayesian models will enable results to be interpreted more intuitively through reporting subjective probabilities rather than null hypothesis tests or frequentist confidence intervals [27]. Each of the primary and additional outcomes will be extracted and analysed as continuous measures. For primary outcomes, the effect of each study will be estimated by calculating the pre-post raw scale mean difference between intervention and control. Modelling outcomes on the same absolute scale as the original measurement will provide more interpretable findings. Effect size estimates for additional outcomes will be standardised using reported standard deviations to account for differences in measurement scales. Standard threshold values of 0.2, 0.5, and 0.8 will be used to describe effect size estimates as small, medium, and large, respectively [28]. Values between 0 and 0.2 will be described as trivial. Three-level Bayesian hierarchical models will be used to pool effect sizes and model average effect, variance within studies, variance between studies, and covariance of multiple outcomes reported in the same study (e.g. single outcome variable reported at intermediate testing points). Informative priors will be used to estimate within-study variances and account for unknown correlations between pre and post values of the different outcomes. Non-informative priors will be used for all other model parameters. Inconsistency in models will be described by comparing variances across the three levels. Inferences from all analyses will be performed on posterior samples generated by Hamiltonian Markov chain Monte Carlo and through the use of credible intervals and calculated probabilities. Analyses will be performed using the R wrapper package brms interfaced with Stan to perform sampling [29].

Sensitivity analyses will be performed to examine the robustness of the outcomes by excluding studies at high risk of bias. Animal and human studies will be aggregated if sufficient data are available and meta-regressions indicate no substantive difference in the median pooled effect size estimate. Meta-regressions where possible will be used to explore the effect of type of supplementation (carnosine or β-alanine), duration of supplementation, and the disease type. Meta-regressions using categorical variables will be performed where there are at least four data points for each factor level.

### Meta-biases

#### Outcome reporting bias

Where possible, we will screen clinical trial registers to compare outcomes reported in the protocol with each published report. Where there is no preregistration or protocol, we will compare the outcomes reported in the methods and results section of each published report.

#### Small study bias (includes publication and study quality bias)

We will visually inspect funnel plots and assess using a multi-level extension of Egger’s regression test [30].

### Confidence in cumulative evidence

The quality of evidence for each outcome will be assessed using the Grading of Recommendations Assessment, Development and Evaluation (GRADE) approach [31], which assesses quality across five domains: risk of bias, inconsistency (heterogeneity), indirectness, imprecision, and publication bias. Quality will be graded as “high”, “moderate”, “low”, or “very low” (Table 2). Outcomes from randomised controlled trials begin as “high” quality evidence and can be downgraded for issues in each domain. Outcomes can also be upgraded when there is evidence of a large magnitude of effect, presence of a dose-response gradient, and all plausible confounders of other biases increase the confidence in the estimated effect [33]. The approach and procedures will be the same as for study selection and data extraction.

**Table 2:** Significance of the four levels of evidence [32]

Quality level	Definition
High	We are very confident that the true effect lies close to that of the estimate of the effect.
Moderate	We are moderately confident in the effect estimate: the true effect is likely to be close to the estimate of the effect, but there is a possibility that it is substantially different.
Low	Our confidence in the effect estimate is limited: the true effect may be substantially different from the estimate of the effect.
Very low	We have very little confidence in the effect estimate: the true effect is likely to be substantially different from the estimate of the effect.

## Discussion

This systematic review and meta-analysis will synthesise evidence to determine the effect of carnosine or β-alanine supplementation on markers of glycaemic control and insulin resistance. By including all available human and animal data, we will provide the most comprehensive overview on the topic to date. Further, the proposed meta-analysis will explore the factors associated with positive outcomes and highlight promising avenues of future research. The results will have implications for those working in prediabetes, diabetes, and metabolic health in general and may lead to the development of new treatment approaches.

## Dissemination

The study results will be presented at a professional conference and published in a peer-reviewed journal.

## Supplementary Information

**Additional file 1.** Supporting information.

## Data Availability

Not applicable.

## Abbreviations

BMI: Body mass index
FG: Fasting glucose
GRADE: Grading of Recommendations Assessment, Development and Evaluation
GTT: Glucose tolerance test
HbA_1c_: Glycated haemoglobin A_1c_
HCD: Histidine-containing dipeptide
MeSH: Medical Subject Headings
PRESS: Peer Review of Electronic Search Strategies
PRISMA: Preferred Reporting Items for Systematic Review and Meta-Analysis
RCTs: Randomised controlled trials
SYRCLE: Systematic Review Centre for Laboratory Animal Experimentation
WHO: World Health Organization
