# Supplementary material for: The effect of carnosine or β-alanine supplementation on markers of glycaemic control and insulin resistance in human and animal studies: a protocol for a systematic review and meta-analysis
Source: Syst Rev. 2020 Dec 5;9:282. doi: 10.1186/s13643-020-01539-8 (PMC7719243; doi:10.1186/s13643-020-01539-8)
Supplement: Supplementary file 1 — Additional file 1. Supporting information. [file 13643_2020_1539_MOESM1_ESM.docx]

**Supporting Information**

**The effect of carnosine or β-alanine supplementation on markers of glycaemic control and insulin resistance in human and animal studies: a protocol for a systematic review and meta-analysis**

Joseph J Matthews, Eimear Dolan, Paul A Swinton, Livia Santos, Guilherme G Artioli, Mark D Turner, Kirsty J Elliott-Sale, Craig Sale

**Contents**

1. Completed PRISMA-P 2015 checklist
2. List of additional outcomes for glycaemic control and insulin resistance
3. Preliminary PubMed search strategy
4. PRISMA flow diagram template
5. **Completed PRISMA-P 2015 checklist**

# **This checklist has been adapted for use with systematic review protocol submissions to BioMed Central journals from Table 3 in Moher D et al:** Preferred reporting items for systematic review and meta-analysis protocols (PRISMA-P) 2015 statement. *Systematic Reviews* 2015 **4**:1

# An Editorial from the Editors-in-Chief of *Systematic Reviews* details why this checklist was adapted - **Moher D, Stewart L & Shekelle P:** Implementing PRISMA-P: recommendations for prospective authors. *Systematic Reviews* 2016 **5**:15

| **Section/topic** | **#** | **Checklist item** | **Information reported** | | **Line number(s)** |
| --- | --- | --- | --- | --- | --- |
|  |  |  | **Yes** | **No** |  |
| **ADMINISTRATIVE INFORMATION** | | | | | |
| **Title** | | | | | |
| Identification | 1a | Identify the report as a protocol of a systematic review |  |  | Title Page |
| Update | 1b | If the protocol is for an update of a previous systematic review, identify as such |  |  | N/A |
| **Registration** | 2 | If registered, provide the name of the registry (e.g., PROSPERO) and registration number in the Abstract |  |  | Line 29 |
| **Authors** | | | | | |
| Contact | 3a | Provide name, institutional affiliation, and e-mail address of all protocol authors; provide physical mailing address of corresponding author |  |  | Title Page |
| Contributions | 3b | Describe contributions of protocol authors and identify the guarantor of the review |  |  | Lines 297-301 |
| **Amendments** | 4 | If the protocol represents an amendment of a previously completed or published protocol, identify as such and list changes; otherwise, state plan for documenting important protocol amendments |  |  | N/A |
| **Support** | | | | | |
| Sources | 5a | Indicate sources of financial or other support for the review |  |  | Lines 287-288 |
| Sponsor | 5b | Provide name for the review funder and/or sponsor |  |  | N/A |
| Role of sponsor/funder | 5c | Describe roles of funder(s), sponsor(s), and/or institution(s), if any, in developing the protocol |  |  | N/A |
| **INTRODUCTION** | | | | | |
| **Rationale** | 6 | Describe the rationale for the review in the context of what is already known |  |  | Lines 32-70 |
| **Objectives** | 7 | Provide an explicit statement of the question(s) the review will address with reference to participants, interventions, comparators, and outcomes (PICO) |  |  | Lines 70-72 |
| **METHODS** | | | | | |
| **Eligibility criteria** | 8 | Specify the study characteristics (e.g., PICO, study design, setting, time frame) and report characteristics (e.g., years considered, language, publication status) to be used as criteria for eligibility for the review |  |  | Lines 80-87 & Table 1. |
| **Information sources** | 9 | Describe all intended information sources (e.g., electronic databases, contact with study authors, trial registers, or other grey literature sources) with planned dates of coverage |  |  | Lines 89-101 |
| **Search strategy** | 10 | Present draft of search strategy to be used for at least one electronic database, including planned limits, such that it could be repeated |  |  | Lines 103-112 & Supporting Information |
| ***STUDY RECORDS*** | | | | | |
| Data management | 11a | Describe the mechanism(s) that will be used to manage records and data throughout the review |  |  | Lines124-126. |
| Selection process | 11b | State the process that will be used for selecting studies (e.g., two independent reviewers) through each phase of the review (i.e., screening, eligibility, and inclusion in meta-analysis) |  |  | Lines 114-118 & 131-137 |
| Data collection process | 11c | Describe planned method of extracting data from reports (e.g., piloting forms, done independently, in duplicate), any processes for obtaining and confirming data from investigators |  |  | Lines 139-141 |
| **Data items** | 12 | List and define all variables for which data will be sought (e.g., PICO items, funding sources), any pre-planned data assumptions and simplifications |  |  | Lines 143-160 |
| **Outcomes and prioritization** | 13 | List and define all outcomes for which data will be sought, including prioritization of main and additional outcomes, with rationale |  |  | Lines 162-169 |
| **Risk of bias in individual studies** | 14 | Describe anticipated methods for assessing risk of bias of individual studies, including whether this will be done at the outcome or study level, or both; state how this information will be used in data synthesis |  |  | Lines 171-178 |
| ***DATA*** | | | | | |
| **Synthesis** | 15a | Describe criteria under which study data will be quantitatively synthesized |  |  | Lines 180-189 |
|  | 15b | If data are appropriate for quantitative synthesis, describe planned summary measures, methods of handling data, and methods of combining data from studies, including any planned exploration of consistency (e.g., *I* ^2^, Kendall’s tau) |  |  | Lines 189-206 |
|  | 15c | Describe any proposed additional analyses (e.g., sensitivity or subgroup analyses, meta-regression) |  |  | Lines 208-214 |
|  | 15d | If quantitative synthesis is not appropriate, describe the type of summary planned |  |  | N/A |
| **Meta-bias(es)** | 16 | Specify any planned assessment of meta-bias(es) (e.g., publication bias across studies, selective reporting within studies) |  |  | Lines 215-223 |
| **Confidence in cumulative evidence** | 17 | Describe how the strength of the body of evidence will be assessed (e.g., GRADE) |  |  | Lines 225-235 |

1. **List of additional outcomes for glycaemic control and insulin resistance**

**Primary Outcomes**

Glycated haemoglobin (HbA_1c_)

Fasting glucose

Glucose tolerance test: 2-hour glucose value

**Additional Outcomes**

Fasting insulin

C-peptide

Glucose tolerance test (GTT) parameters:

Glucose area under the curve (AUC)

Insulin area under the curve (AUC)

Homeostatic model assessment (HOMA) parameters:

HOMA-IR (insulin resistance)

HOMA-β (beta-cell function)

HOMA-S (insulin sensitivity)

1. **Preliminary PubMed search strategy ­**– <https://pubmed.ncbi.nlm.nih.gov/>

**Search Builder**

1. “insulin” [MeSH Terms]
2. “insulin” [All Fields]
3. “blood glucose” [MeSH Terms]
4. “fasting glucose” [All Fields]
5. “blood glucose” [All Fields]
6. “plasma glucose” [All Fields]
7. “glycemic” [All Fields]
8. “glycaemic” [All Fields]
9. “glycated hemoglobin A” [MeSH Terms]
10. “glycated haemoglobin A” [All Fields]
11. “HbA1c” [All Fields]
12. or/14-24
13. and/10, 13, 25
14. “glucose metabolism disorders” [MeSH Terms]
15. “metabolic syndrome” [MeSH Terms]
16. “diabetes” [All Fields]
17. “prediabetes” [All Fields]
18. “hyperglycemia” [All Fields]
19. “hyperglycaemia” [All Fields]
20. “overweight” [MeSH Terms]
21. “overweight” [All Fields]
22. “obesity” [All Fields]
23. or/1-9
24. “carnosine” [Title/Abstract]
25. “beta-alanine” [Title/Abstract]
26. or/11-12

Filters or Limiters: None applied.

| **Overview** | |
| --- | --- |
| Full Search | ((“glucose metabolism disorders” [MeSH Terms] OR “metabolic syndrome” [MeSH Terms] OR “diabetes” [All Fields] OR “prediabetes” [All Fields] OR “hyperglycemia” [All Fields] OR “hyperglycaemia” [All Fields] OR “overweight” [MeSH Terms] OR “overweight” [All Fields] OR “obesity” [All Fields]) AND ("carnosine" OR "beta-alanine"[Title/Abstract])) AND (“insulin” [MeSH Terms] OR “insulin” [All Fields] OR “blood glucose” [MeSH Terms] OR “fasting glucose” [All Fields] OR “blood glucose” [All Fields] OR “plasma glucose” [All Fields] OR “glycemic” [All Fields] OR “glycaemic” [All Fields] OR “glycated hemoglobin A” [MeSH Terms] OR “glycated haemoglobin A” [All Fields] OR “HbA1c” [All Fields]) |
| **Breakdown** | |
| Population | “glucose metabolism disorders” [MeSH Terms] OR “metabolic syndrome” [MeSH Terms] OR “diabetes” [All Fields] OR “prediabetes” [All Fields] OR “hyperglycemia” [All Fields] OR “hyperglycaemia” [All Fields] OR “overweight” [MeSH Terms] OR “overweight” [All Fields] OR “obesity” [All Fields] |
| Intervention | "carnosine" OR "beta-alanine"[Title/Abstract] |
| Outcome | “insulin” [MeSH Terms] OR “insulin” [All Fields] OR “blood glucose” [MeSH Terms] OR “fasting glucose” [All Fields] OR “blood glucose” [All Fields] OR “plasma glucose” [All Fields] OR “glycemic” [All Fields] OR “glycaemic” [All Fields] OR “glycated hemoglobin A” [MeSH Terms] OR “glycated haemoglobin A” [All Fields] OR “HbA1c” [All Fields] |

1. **PRISMA flow diagram depicting the search process**

**Screening**

**Included**

**Eligibility**

**Identification**

Records identified through electronic

databases (n = )

PubMed (n = ) CENTRAL (n = )

Scopus (n =) Web of Science (n = )

ProQuest (n = ) CINAHL Complete (n= )

Records after duplicates removed
(n = )

Records excluded
(n = )

Full-text articles excluded, with reasons (n = )

Inappropriate population (n = )

Inappropriate intervention (n = )

Inappropriate comparator (n = )

Inappropriate outcome (n = )

Inappropriate study design (n = )

Multiple inappropriate criteria (n = )

Other (n = )

Additional records identified through other sources

(n = )

Articles included in

meta-analysis
(n = )

Records screened based on title and abstract (n = )

Full text articles assessed for eligibility (n = )
